# Gastrointestinal symptoms in low-dose aspirin users: a comparison between plain and buffered aspirin

**DOI:** 10.1007/s12471-014-0522-3

**Published:** 2014-02-13

**Authors:** J. Jaspers Focks, M. M. Tielemans, L. G. M. van Rossum, T. Eikendal, M. A. Brouwer, J. B. M. J. Jansen, R. J. F. Laheij, F. W. A. Verheugt, M. G. H. van Oijen

**Affiliations:** 1 Department of Cardiology, Radboud University Nijmegen Medical Center, 670, Geert Grooteplein Zuid 10, 6525 GA Nijmegen, the Netherlands; 2 Department of Gastroenterology and Hepatology, Radboud University Nijmegen Medical Center, Nijmegen, the Netherlands; 3 Department of Health Evidence, Radboud University Nijmegen Medical Center, Nijmegen, the Netherlands; 4 Department of Gastroenterology, Elkerliek Hospital, Helmond, the Netherlands; 5 Department of Gastroenterology, University Medical Center Utrecht, Utrecht, the Netherlands; 6 Division of Digestive Diseases, David Geffen School of Medicine at UCLA, Los Angeles, CA USA

**Keywords:** Low-dose aspirin, Effervescent calcium carbasalate, Gastrointestinal symptoms, Survey, Community based population

## Abstract

**Background:**

Aspirin is associated with gastrointestinal side effects such as gastric ulcers, gastric bleeding and dyspepsia. High-dose effervescent calcium carbasalate (ECC), a buffered formulation of aspirin, is associated with reduced gastric toxicity compared with plain aspirin in healthy volunteers, but at lower cardiovascular doses no beneficial effects were observed.

**Aim:**

To compare the prevalence of self-reported gastrointestinal symptoms between low-dose plain aspirin and ECC.

**Methods:**

A total of 51,869 questionnaires were sent to a representative sample of the Dutch adult general population in December 2008. Questions about demographics, gastrointestinal symptoms in general and specific symptoms, comorbidity, and medication use including bioequivalent doses of ECC (100 mg) and plain aspirin (80 mg) were stated. We investigated the prevalence of self-reported gastrointestinal symptoms on ECC compared with plain aspirin using univariate and multivariate logistic regression analyses.

**Results:**

A total of 16,715 questionnaires (32 %) were returned and eligible for analysis. Of these, 911 (5 %) respondents reported the use of plain aspirin, 633 (4 %) ECC and 15,171 reported using neither form of aspirin (91 %). The prevalence of self-reported gastrointestinal symptoms in general was higher in respondents using ECC (27.5 %) compared with plain aspirin (26.3 %), but did not differ significantly with either univariate (OR 1.06, 95 %CI 0.84–1.33), or multivariate analysis (aOR 1.08, 95 %CI 0.83–1.41). Also, none of the specific types of symptoms differed between the two aspirin formulations.

**Conclusions:**

In this large cohort representative of the general Dutch population, low-dose ECC is not associated with a reduction in self-reported gastrointestinal symptoms compared with plain aspirin.

**Electronic supplementary material:**

The online version of this article (doi:10.1007/s12471-014-0522-3) contains supplementary material, which is available to authorized users.

## Introduction

Optimal antithrombotic therapy has proven to be essential in secondary prevention in cardiovascular disease. In this, aspirin has a pivotal role and is associated with a relative reduction of approximately 25 % in recurrent cardiovascular events [1]. However, gastric toxicity is a well-known side effect of aspirin presenting as gastric or duodenal ulcers, bleeding and dyspepsia [1–7]. Of these, dyspepsia is most often reported (in 20–40 % of chronic aspirin users) [4, 7, 8] and is associated with reduced aspirin compliance [9, 10], increased healthcare costs [11] and reduced health-related quality of life [12].

To reduce gastrointestinal damage, different formulations of aspirin have been developed. These formulations either delay the release of aspirin beyond the stomach (enteric-coated aspirin), facilitate the transit of aspirin across the gastric mucous layer (PL2200), or increase solubility of aspirin supposedly resulting in lower irritating concentrations on the gastric mucosa (effervescent calcium carbasalate (ECC)). The gastric toxicity of different formulations was mainly studied in high dosages and showed clear benefit over plain aspirin with respect to gastric ulcer formation when studied in healthy volunteers [13–18]. However, when investigating its clinical effect in patients on (low-dose) chronic antiplatelet therapy, no clear beneficial effect on gastrointestinal side effects was noticeable [19–22].

In the Netherlands, a total of 1,290,000 patients use low-dose aspirin of which 41 % are prescribed ECC [23]. No data have been published comparing the effects of ECC and plain aspirin on gastrointestinal symptoms. In this population-based cohort of respondents using low-dose aspirin we studied and compared the prevalence of gastrointestinal symptoms between those using ECC and plain aspirin. We also studied whether respondents using different formulations may report different types of gastrointestinal symptoms.

## Methods

### Study population

We sent 51,869 questionnaires by surface mail to a representative sample of the Dutch population in December 2008. Invited subjects were aged 18 years and above, and randomly selected from municipal databases of five different municipalities selected on their geographical location in the Netherlands, in order to gather a representative sample of the Dutch population. We included returned questionnaires until 31 March 2009. We excluded returned questionnaires with missing elements that were part of the primary outcome measure. We also excluded returned questionnaires in which all baseline characteristics were missing or when the medication was unreadable or if the used aspirin formulation was not reported. The complete cohort has been described previously [24]. The current sample size consisted of those respondents reporting the use of either low-dose plain aspirin or ECC.

The Medical Ethical Committee of the Radboud University Nijmegen assessed the research proposal of this study and concluded that it could be waived for ethical review, as questionnaires were returned and stored anonymously, and (non-)responders would not be contacted again. For this reason, we did not obtain written informed consent.

### Questionnaire

The questionnaire has been used before and was specifically designed for collection of demographic information, gastrointestinal symptoms, and medication use [25, 26]. Participants were asked whether they suffer from gastrointestinal symptoms in general and about the presence of 26 gastrointestinal symptoms such as nausea, early satiety and bloating. Severity of gastrointestinal symptoms was assessed on a seven-point Likert scale (0 = absent, 1 = almost absent, 2 = mild, 3 = moderate, 4 = moderately severe, 5 = severe, 6 = very severe) over the preceding 4 weeks [27]. A symptom was considered to be present if the participants scored ≥2 on the Likert scale.

### Outcomes

Our primary outcome was the presence of gastrointestinal symptoms, which was assessed with the question: ‘Do you experience gastrointestinal complaints?’ and had to be answered with either ‘yes’ or ‘no’. Secondary outcomes were duration of the primary endpoint and the individual gastrointestinal symptoms among responders who reported the presence of gastrointestinal complaints. The primary and secondary outcomes were compared between respondents reporting the use of low-dose plain aspirin (80 mg) and those using ECC (100 mg).

### Statistical analysis

Statistical analyses were performed with SPSS statistical software, version 16.0 (SPSS, Inc., Chicago, Illinois, USA). Frequency tables were provided describing respondents’ baseline characteristics. Pearson’s chi squared test was used to compare categorical variables. Continuous variables were compared with the Student *T*-test or the Mann–Whitney U method whenever appropriate. Univariate and multivariate associations for gastrointestinal endpoints in respondents using plain aspirin or ECC were analysed using logistic regression. Two-sided p-values of <0.05 were considered statistically significant. Covariates were included in the multivariate analysis if they significantly differed between respondents using ECC versus plain aspirin. In addition, those covariates associated with gastrointestinal symptoms at a level of *p* < 0.1 in univariate analysis were included in multivariate analysis. Using forward selection, a covariate was allowed into the multivariate model if it influenced the model with a likelihood ratio significance level of *p* < 0.05, and was removed again if its significance level exceeded *p* = 0.1 during any of the following steps. The type of formulation used (ECC versus plain aspirin) was forced into the model.

With respect to the duration of symptoms and the analyses of the individual symptoms, only participants reporting the presence of the primary outcome were selected. The duration of symptoms was compared using the Mann–Whitney U method. The individual symptoms were divided into upper and lower gastrointestinal symptoms and figures were provided describing their frequencies. The sum of the individual symptoms present was categorised according to the number of symptoms present and frequencies were provided.

## Results

**Fig. 1:**
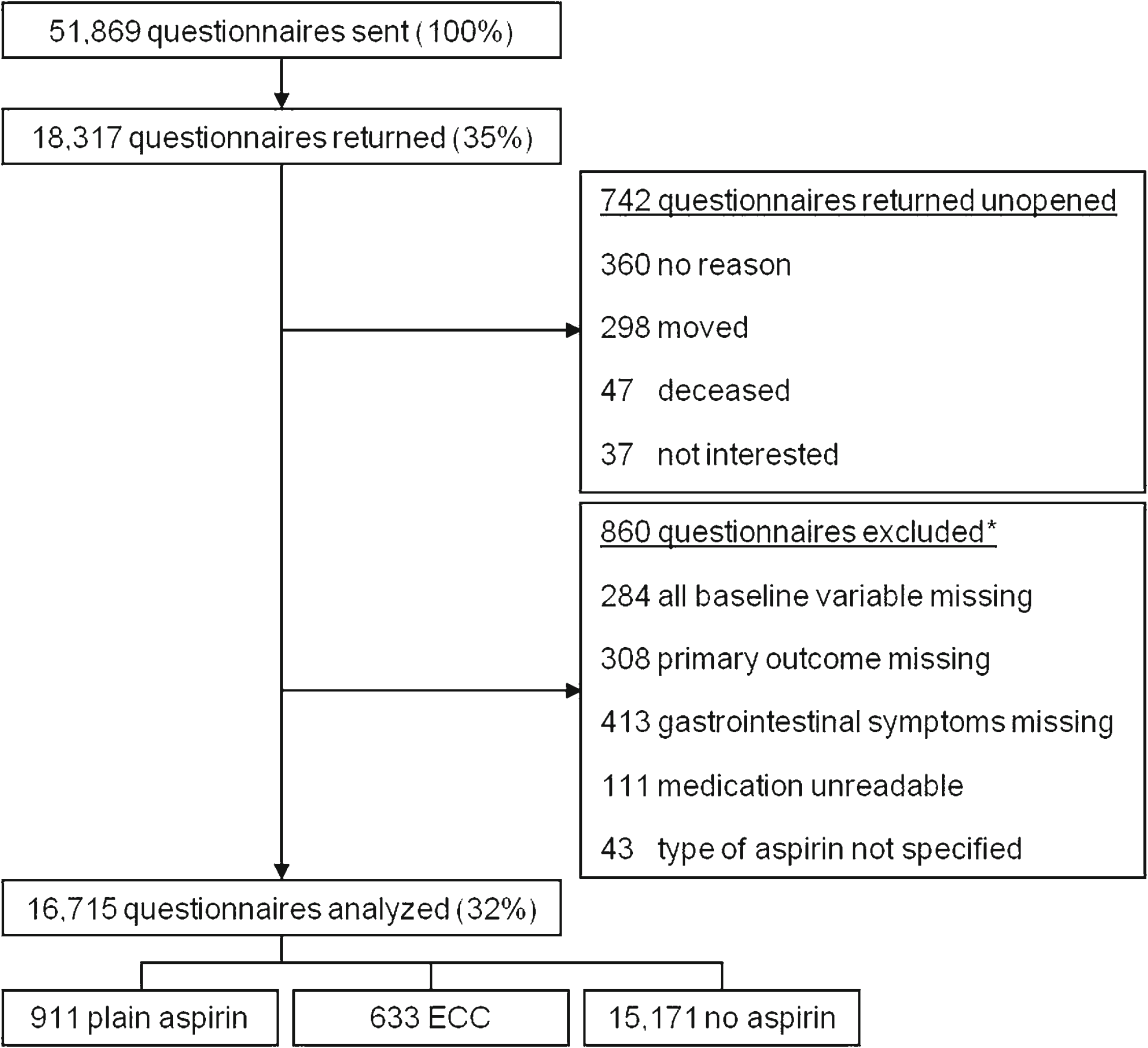
Flow chart. *Some respondents fulfilled more than 1 exclusion criterion

A total of 18,317 (35 %) questionnaires were returned, of which 742 unopened for various reasons (Fig. 1). After applying our predetermined exclusion criteria a total of 16,715 questionnaires were included in our analyses. In total, 911 persons (5.4 %) reported plain aspirin use, 633 ECC (3.8 %) and 15,171 reported not using any form of aspirin (90.8 %). Nearly all baseline characteristics differed between participants with and without aspirin (Supplementary Table 1). When comparing plain aspirin and ECC, participants using ECC were older, reported more comorbidity and were using more concomitant medication (Table 1).

**Table 1:** Baseline characteristics

	Plain aspirin	Effervescent calcium carbasalate	*P*-value
	*N* = 911	*N* = 633	
Mean age (±SD) (years)	59.7 (15.2)	64.7 (11.3)	<0.01
Male (%)	494 (56)	377 (61)	0.09
Smoking (%)	160 (18)	116 (19)	0.74
BMI (±SD) (kg/m^2^)	26.3 (4.6)	27.0 (4.9)	<0.01
Comorbidity (%)			
Diabetes mellitus	108 (12)	106 (17)	<0.01
Rheumatoid arthritis	53 (6)	54 (9)	0.04
Asthma/COPD	62 (7)	69 (11)	<0.01
Coeliac disease	16 (2)	9 (1)	0.61
IBD	27 (3)	18 (3)	0.89
Medication use (%)			
PPI	191 (21)	188 (30)	<0.01
H2RA	24 (3)	14 (2)	0.60
Antacids	79 (9)	50 (8)	0.59
Paracetamol	474 (52)	276 (44)	<0.01
NSAIDs	274 (30)	186 (29)	0.77
Clopidogrel	17 (2)	36 (6)	<0.01
Dipyridamole	43 (5)	69 (11)	<0.01
Beta blockers	351 (39)	301 (48)	<0.01
ACE inhibitors	175 (19)	189 (30)	<0.01
Angiotensin-receptor antagonist	103 (11)	83 (13)	0.28
Calcium antagonist	128 (14)	105 (17)	0.17
Diuretics	185 (20)	155 (25)	0.051
Statins	396 (44)	373 (59)	<0.01
Systemic corticosteroids	15 (2)	11 (2)	0.89
Oral glucose lowering agents	85 (9)	70 (11)	0.27
Antidepressants	47 (5)	40 (6)	0.33
History (%)			
Peptic ulcer disease	69 (8)	76 (12)	<0.01
Peptic ulcer bleeding	26 (3)	15 (2)	0.56

*SD* standard deviation, *BMI* body mass index, kg/m^2^ = kilogram per square meter, *COPD* chronic obstructive pulmonary disease, *IBD* inflammatory bowel disease, *PPI* proton pump inhibitor, *H2RA* H2-receptor antagonist, *NSAID* non-steroid anti-inflammatory disease, *ACE* angiotensin converting enzyme

The self-reported prevalence of gastrointestinal symptoms of no aspirin, plain aspirin, and ECC were 25.6 %, 26.3 %, and 27.5 %, respectively. We observed no difference between plain aspirin and ECC for self-reported gastrointestinal symptoms (ECC: OR 1.06, 95 % CI 0.84–1.33). Also after adjustment with multivariate regression for multiple possible confounders there was no significant difference between plain aspirin and ECC for the presence of gastrointestinal symptoms (ECC: aOR 1.08, 95 % CI 0.83–1.41, Table 2). Among those reporting gastrointestinal symptoms, respondents using ECC had a significantly longer history of symptoms (10 years, IQR 4–20) compared with participants using plain aspirin (7 years, IQR 3–16, *p* = 0.04).

**Table 2:** Multivariate logistic regression model for reporting gastrointestinal symptoms with effervescent calcium carbasalate entered into the model

	aOR	95 % CI	*P*-value
Age (per year increase)	0.98	0.97–0.99	<0.01
Male gender	0.71	0.55–0.92	0.01
Comorbidity			
Asthma/COPD	1.54	1.01–2.36	0.046
IBD	2.01	1.00–4.04	0.050
Medication use			
PPI	3.96	2.96–5.30	<0.01
H2RA	4.39	2.01–9.57	<0.01
Antacids	2.90	1.90–4.44	<0.01
Paracetamol	1.42	1.09–1.86	<0.01
Effervescent calcium carbasalate	1.08	0.83–1.41	0.57
History			
Peptic ulcer disease	2.39	1.60–3.58	<0.01

*aOR* adjusted odds ratio, *CI* confidence interval, *COPD* chronic obstructive pulmonary disease, *IBD* inflammatory bowel disease, *PPI* proton pump inhibitor, *H2RA* H2-receptor antagonist

**Fig. 2:**
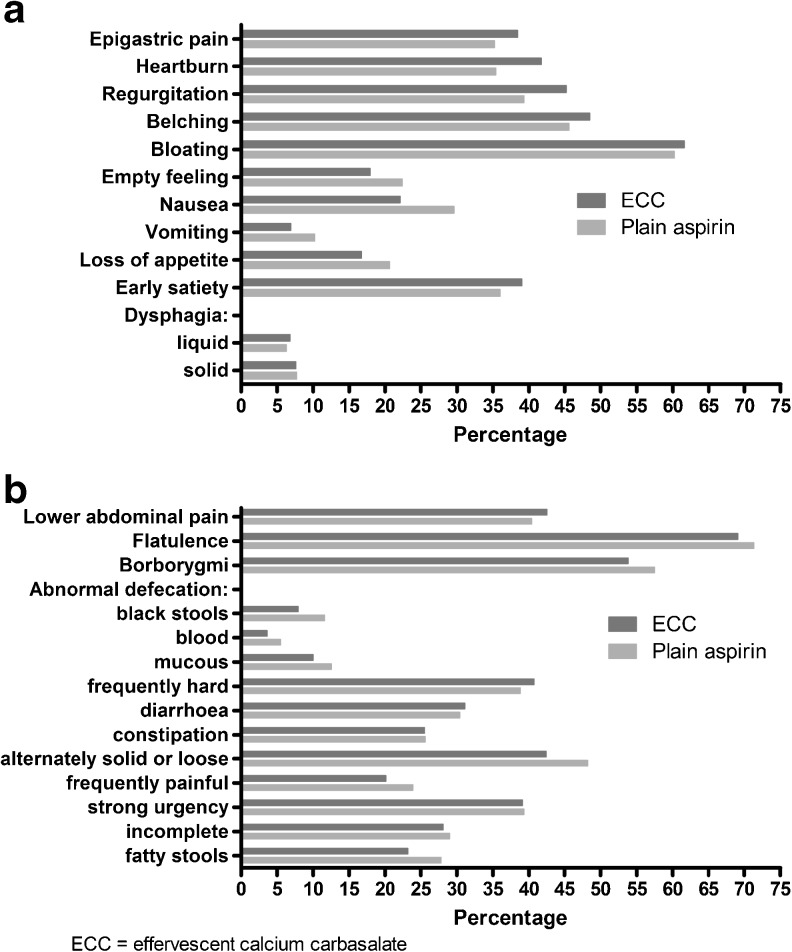
Type and prevalence of specific upper (**a**) and lower (**b**) gastrointestinal symptoms in respondents experiencing gastrointestinal symptoms categorised by aspirin formulation

In respondents reporting the presence of gastrointestinal symptoms and using either plain aspirin or ECC the presence of no more than one individual upper gastrointestinal symptom was reported by 26.9 % while five or more symptoms were reported present by 32.3 %. The most frequently reported upper gastrointestinal symptoms were bloating (61 %), belching (47 %) and regurgitation (42 %) (Fig. 2a). With respect to lower gastrointestinal symptoms, 23.0 % reported no more than one symptom, while 39.0 % experienced the presence of 5 or more symptoms. Flatulence (70 %) and borborygmi (56 %) were the most frequently reported lower gastrointestinal symptoms (Fig. 2b). No significant differences between plain aspirin and ECC were present for any of the upper or lower gastrointestinal symptoms.

## Discussion

We aimed to compare the prevalence of self-reported gastrointestinal symptoms between respondents using plain aspirin and those who were prescribed ECC. We observed that in respondents using any form of low-dose aspirin the prevalence of gastrointestinal symptoms was 27 %. The use of ECC is not associated with less gastrointestinal symptoms compared with plain aspirin. The most reported upper gastrointestinal symptoms were bloating, belching, and regurgitation, whereas flatulence and borborygmi were reported most for lower gastrointestinal symptoms. No differences in the type of symptoms between users of ECC and plain aspirin were observed.

The prevalence of gastrointestinal symptoms in our study cohort is in line with previously reported data of aspirin users [4, 7, 8]. Interestingly, the prevalence of gastrointestinal symptoms in our non-aspirin using population is comparable with those who use aspirin. The selection of our study population could have contributed to this finding. Low-dose aspirin is generally a long-term treatment, i.e. for the remainder of the patients’ life span. For our study we selected all low-dose aspirin users from a large cohort of randomly selected participants returning the questionnaire. As a result of this study design the odds that aspirin treatment was recently initiated for our participants are minimal. Those patients who suffered from gastrointestinal symptoms during (the initiation of) aspirin treatment were likely to receive co-treatment with a proton pump inhibitor, H2-receptor antagonist or antacid or were even switched to other antiplatelet agents. Consequently, our cohort may consist of a selected population of respondents in whom aspirin is relatively well tolerated. This hypothesis is supported by the more frequent use of gastroprotective agents in low-dose aspirin users compared with our non-aspirin using population (e.g. proton pump inhibitor use: 25 % vs 9 %). Irrespectively, our data indicate that ECC is of no beneficial value for gastrointestinal symptoms among our population of long-term aspirin users.

So far, only two studies have been conducted to investigate endoscopically proven gastric mucosal damage in users of ECC and plain aspirin. In a randomised cross-over trial, ECC significantly reduced endoscopically observed gastric erosions and ulcers compared with the bioequivalent dose of plain aspirin [13]. However, this study assessed healthy volunteers, investigated very high doses of aspirin (650 mg three times a day) and only studied the short-term effects. More recently, the effects of low-dose ECC and plain aspirin were compared in patients using long-term aspirin for cardiovascular prevention [19]. In this large retrospective cohort study, the authors concluded that the incidence rates of endoscopically proven peptic ulcers were not significantly different between the two groups.

This is the first study comparing the effects of ECC with plain aspirin for gastrointestinal symptoms. Moreover, in order to obtain a representative sample, persons were randomly selected through databases of local authorities without stringent inclusion and exclusion criteria. We do acknowledge some limitations in our study. First, due to our study design, response bias could be a potential limitation. Due to concealment we were unable to contact non-responders and compare their characteristics with responders. To minimise the effect of response bias all participants were invited with a personalised invitational letter and were asked explicitly to participate irrespective of experiencing gastrointestinal symptoms. Seventy-five percent of all respondents indeed did not report the presence of gastrointestinal symptoms. Secondly, we did not study the duration of low-dose aspirin use or the effect of gastrointestinal symptoms on aspirin compliance. Finally, we observed important differences in baseline characteristics between the two aspirin formulations, all to the detriment of those participants using ECC. This could be an indication that physicians are more likely to prescribe plain aspirin to the relatively healthy subjects and preferentially prescribe ECC to the older and more fragile patients. In order to adjust for this possible bias we performed multivariate analysis. Nonetheless, this observation suggesting confounding by indication should be noted and calls for a study with random allocation of aspirin formulation.

We observed that low-dose ECC is not associated with a reduction in gastrointestinal symptoms compared with plain aspirin. This absence of a beneficial effect of ECC over plain aspirin is in analogy to a previous study indicating that low-dose ECC is not associated with a reduction in the prevalence of gastric ulcer complications. Notably, the costs of ECC are significantly higher compared with plain aspirin (€1.55 vs. €0.79/month in the Netherlands) [28]. With 530,000 ECC users in the Netherlands, these additive costs comprise nearly 5 million euro annually. In view of the lack of a beneficial clinical effect and the higher costs of ECC, we feel that plain aspirin is the first drug of choice. If gastrointestinal symptoms occur, we advise to prescribe a relatively cheap proton pump inhibitor with proven beneficial effects [29–31]. Only if this does not reduce the symptoms, might one consider ECC as an alternative to plain aspirin.

In conclusion, the prevalence of gastrointestinal symptoms among aspirin users in the Dutch community is 27 % with no difference between effervescent calcium carbasalate and plain aspirin in overall prevalence and type of symptoms reported.

## Electronic supplementary material

Below is the link to the electronic supplementary material.

Supplementary Table 1 (DOCX 29 kb)
