# Supplementary material for: Gastrointestinal symptoms in low-dose aspirin users: a comparison between plain and buffered aspirin
Source: Neth Heart J. 2014 Feb 13;22(3):107–12. doi: 10.1007/s12471-014-0522-3 (PMC3931859; doi:10.1007/s12471-014-0522-3)
Supplement: Supplementary file 1 — (DOCX 29 kb) [file 12471_2014_522_MOESM1_ESM.docx]

**Supplementary Table 1: Baseline characteristics categorised by aspirin use**

|  | | **No aspirin** | **Aspirin** | **P-value** |
| --- | --- | --- | --- | --- |
|  | | **N = 15,171** | **N = 1544** |  |
| Mean age (±SD) (years) | | 48.6 (15.3) | 61.7 (13.9) | <0.01 |
| Male (%) | | 6173 (42) | 871 (58) | <0.01 |
| Smoking (%) | | 2,641 (18) | 276 (18) | 0.71 |
| BMI (±SD) (kg/m^2^) | | 25.2 (4.3) | 26.6 (4.7) | <0.01 |
| Comorbidity (%) | |  |  |  |
|  | Diabetes mellitus | 655 (4) | 214 (14) | <0.01 |
|  | Rheumatoid arthritis | 663 (4) | 107 (7) | <0.01 |
|  | Asthma / COPD | 909 (6) | 131 (9) | <0.01 |
|  | Coeliac disease | 130 (1) | 25 (2) | <0.01 |
|  | IBD | 259 (2) | 45 (3) | <0.01 |
| Medication use (%) | |  |  |  |
|  | PPI | 1380 (9) | 379 (25) | <0.01 |
|  | H2RA | 245 (2) | 38 (3) | 0.01 |
|  | Antacids | 886 (6) | 129 (8) | <0.01 |
|  | Paracetamol | 7513 (50) | 750 (49) | 0.48 |
|  | NSAIDs | 2758 (18) | 460 (30) | <0.01 |
|  | Clopidogrel | 31 (0.2) | 53 (3) | <0.01 |
|  | Dipyridamole | 12 (0.1) | 112 (7) | <0.01 |
|  | Beta blockers | 1104 (7) | 652 (42) | <0.01 |
|  | ACE inhibitors | 577 (4) | 364 (24) | <0.01 |
|  | Angiotensin-receptor antagonists | 512 (3) | 186 (12) | <0.01 |
|  | Calcium antagonist | 338 (2) | 233 (15) | <0.01 |
|  | Diuretics | 1095 (7) | 340 (22) | <0.01 |
|  | Statins | 932 (6) | 769 (50) | <0.01 |
|  | Systemic corticosteroids | 112 (1) | 26 (2) | <0.01 |
|  | Oral glucose lowering agents | 424 (3) | 155 (10) | <0.01 |
|  | Antidepressants | 612 (4) | 87 (6) | <0.01 |
| History (%) | |  |  |  |
|  | Peptic ulcer disease | 736 (5) | 145 (10) | <0.01 |
|  | Peptic ulcer bleeding | 240 (2) | 41 (3) | <0.01 |

SD = standard deviation, BMI = body mass index, kg/m^2^ = kilogram per square meter, COPD = chronic obstructive pulmonary disease, IBD = inflammatory bowel disease, PPI = proton pump inhibitor, H2RA = H2-receptor antagonist, NSAID = non-steroid anti-inflammatory disease, ACE = angiotensin converting enzyme.
